# Supplementary material for: Coherent characterisation of a single molecule in a photonic black box
Source: Nat Commun. 2021 Jan 29;12:706. doi: 10.1038/s41467-021-20915-z (PMC7846597; doi:10.1038/s41467-021-20915-z)
Supplement: Supplementary file 1 — Supplementary Information [file 41467_2021_20915_MOESM1_ESM.pdf]

Supplementary Information for:
Coherent characterisation of a single molecule in a photonic black box

Sebastien Boissier,[1] Ross C. Schofield,[1] Lin Jin,[2] Anna Ovvyan,[2] Salahuddin Nur,[1] Frank H. L. Koppens,[3] Costanza Toninelli,[4] Wolfram H. P. Pernice,[2] Kyle D. Major,[1] E. A. Hinds,[1] and Alex S. Clark[1,*]

[1] *Centre for Cold Matter, Blackett Laboratory, Imperial College London, Prince Consort Road, SW7 2AZ, London, United Kingdom*
[2] *Physikalisches Institut, Westfälische Wilhelms, Universität Münster, Heisenbergstrasse 11, 48149 Münster, Germany*
[3] *ICFO – Institut de Ciencies Fotoniques, The Barcelona Institute of Science and Technology, 08860 Castelldefels (Barcelona), Spain*
[4] *LENS and CNR-INO, Via Nello Carrara 1, 50019 Sesto Fiorentino (FI), Italy*

(Dated: December 18, 2020)

CONTENTS

List of Figures 1

I. Analytical solutions for simple geometries 1
A. Continuous waveguide 1
B. Symmetrical cavity in the weak-coupling regime 2

II. Coupling efficiency simulations 3

III. Micro-fluidic channel filling 3

IV. Imperfect filtering of off-resonant light 4

References 4

LIST OF FIGURES

S1 FDTD simulations to explore the coupling efficiency in our device. 5
S2 Sketch of the fluorescence microscopy set-up 6
S3 Characterisation of filled micro-fluidic channels 7

I. ANALYTICAL SOLUTIONS FOR SIMPLE GEOMETRIES

For structures where a normal mode decomposition is appropriate, we can compare Eq. 9 and Eq. 10 of the main text with analytical solutions for the Green function, or with results of coupled-mode theory, to obtain expressions that depend explicitly on structural parameters. We do this for the case of a single-mode continuous waveguide and for a cavity in the weak-coupling regime.

A. Continuous waveguide

For a continuous waveguide with no loss, $|r_0| = 0$ and $|t_0| = 1$. In that case, the analytical solution for the Green function [1] allows us to write Eq. 6 of the main text as [2, 3]

* Email: alex.clark@imperial.ac.uk

$$\mathcal{E}_{\text{out}} = \left(1 + i\frac{\beta_{\text{g}}\gamma_1}{\Omega}\sigma^-\right)|\mathcal{E}_{\text{in}}|\,, \tag{S1}$$

where $\beta_{\text{g}} = 2\beta_{\text{pump}} = 2\beta_{\text{probe}}$. This is the same result as we would have obtained by applying bosonic annihilation and creation operators to the waveguide modes [4, 5]. Comparison of Eq. S1 with Eq. 6 of the main text shows that $\phi_{\text{T}} = \pi/2$ and hence that the normalised transmission given by Eq. 9 of the main text is

$$\frac{P_{\text{out}}}{P_{\text{in}}} = 1 - \alpha\beta_{\text{g}}\left(2 - \alpha\beta_{\text{g}}\right)\frac{\Gamma_1/(2\Gamma_2)}{(\delta\omega/\Gamma_2)^2 + 1 + S}\,. \tag{S2}$$

The same expression can be found in [3]. Because $|r_0| = 0$, the reflection spectrum given by Eq. 10 of the main text becomes

$$\frac{P_{\text{refl}}}{P_{\text{in}}} = (\alpha\beta_{\text{g}})^2\frac{\Gamma_1/(2\Gamma_2)}{(\delta\omega/\Gamma_2)^2 + 1 + S}\,. \tag{S3}$$

B. Symmetrical cavity in the weak-coupling regime

Let us now consider a waveguide interrupted by a symmetrical cavity, whose mode is matched to the transverse guide mode. In the absence of an emitter the transmission coefficient for the field is (see, e.g. [6])

$$t_0 = \frac{-1}{1 - i(\omega - \omega_C)/\kappa}\,, \tag{S4}$$

where ω_c is the resonant frequency of the cavity and κ is the damping rate for the cavity field decaying freely into the two waveguides. When the emitter is pumped by light that is resonant with the cavity, we call the (partial) radiation rate of the excited emitter into the cavity mode γ_{cav}. In general, the cavity is not resonant with the pump light and then the rate decreases to $|t_0|^2\gamma_{\text{cav}}$ [7]. Hence, in the language of the main text

$$\beta_{\text{cav}}\gamma_1 = |t_0|^2\gamma_{\text{cav}}\,, \tag{S5}$$

where $\beta_{\text{cav}} = 2\beta_{\text{pump}} = 2\beta_{\text{probe}}$. Neglecting the quantum noise of the field, as in the main text, the literature [6, 8] gives the following relation between the input and output fields in the weak coupling regime:

$$\mathcal{E}_{\text{out}} = t_0\mathcal{E}_{\text{in}} + i\sqrt{\frac{\gamma_{\text{cav}}}{2}}t_0\sigma^-\,. \tag{S6}$$

Reference [8] also gives the steady-state solution for the Rabi frequency as

$$\Omega = -2t_0\mathcal{E}_{\text{in}}\sqrt{\frac{\gamma_{\text{cav}}}{2}}\,. \tag{S7}$$

We substitute Eq. S7 and Eq. S5 into Eq. S6 to find, neglecting an over-all phase, that

$$\mathcal{E}_{\text{out}} = \left(|t_0| - i\frac{t_0}{|t_0|}\frac{\beta_{\text{cav}}\gamma_1}{\Omega}\sigma^-\right)|\mathcal{E}_{\text{in}}|\,, \tag{S8}$$

On comparing this result with Eq. 6 of the main text, we see that $\phi_{\text{T}} = \pi/2 + \text{Arg}(-t_0)$, which depends on the scaled detuning from the cavity resonance $(\omega - \omega_C)/\kappa$, but not on the position of the emitter, as also noted in [6]. Following [6] this analysis is readily extended to lossy and non-symmetric cavities.

II. COUPLING EFFICIENCY SIMULATIONS

We used finite-difference, time-domain (FDTD) calculations to explore the behaviour of the device and to predict values for the coupling efficiency $\beta_{\rm probe}$ and phase shift $\phi_{\rm T}$ at the ZPL frequency of DBT. The following simulation results are obtained with the channel height and under-etch (defined in Fig. 2(c) of the main text) fixed at 1 µm and 150 nm respectively, which are the values used in the experiment. The channel width is set at the gap length + 200 nm to allow for the uncertainty in alignment. Fig. S1(a-c) show simulation results for a dipole sitting in the middle of the gap on the centre line of the waveguides, and polarised along x. In Fig. S1(a) we show the coupling efficiency $\beta_{\rm probe}$ for a waveguide 200 nm high and 400 nm wide, these being the dimensions used in our experiment. We see that $\beta_{\rm probe}$ decreases rapidly with the length of the gap and conclude that a short gap is necessary for good coupling. On the other hand, we know that the molecule loses spectral stability if it is less than one or two hundred nm from the interface at the end of the guide, so we consider a good gap length may be 300 nm. The same graph also shows the Purcell factor $\gamma_1/\gamma_{1,\rm free}$, where $\gamma_{1,\rm free}$ is the value of γ_1 when the dipole is in homogeneous anthracene. This factor stays close to 1. We also checked at the frequencies corresponding to the strongest vibrational lines of DBT and find that there is no significant change. We therefore conclude that γ_1 and α are unaltered by the dielectric environment.

Fixing the gap length at 300 nm, we plot $\beta_{\rm probe}$ in Fig. S1(b) as a function of the waveguide height and width. As the size of the guide increases, so does $\beta_{\rm probe}$; indeed, this plot shows that the coupling efficiency found in Fig. S1(a) could be improved by increasing the waveguide height to 350 nm or 400 nm. We can understand this behaviour by noting that a larger guided mode diffracts less strongly in the gap and this reduced angular spread increases the overlap of the mode field with the field of the dipole, resulting in an increase in $\beta_{\rm probe}$. However, if we continue to increase the size of the guide, the increasing spatial spread of the mode starts to reduce the overlap and $\beta_{\rm probe}$ eventually declines again. In Fig. S1(b) the guide becomes multi-mode before the maximum of $\beta_{\rm probe}$ is reached, and that is the region shown in pink. In this experiment we do not want to operate in the multi-mode regime.

In Fig. S1(c), we show for several different gap lengths the waveguide dimensions that maximise $\beta_{\rm probe}$. With a 300 nm gap the maximum $\beta_{\rm probe}$ is 17.7% and this lies in the multi-mode regime. On reducing the gap to 100 nm the maximum of $\beta_{\rm probe}$ moves into the single-mode regime and increases to 20.9%, but in practice this gap is too small to expect the molecule to be spectrally stable.

Finally, Fig. S1(d) shows the variation in $\beta_{\rm probe}$ when we vary the position of the dipole in the transverse (xy) direction so that it no longer sits at the maximum field of the mode in that plane. The guide dimensions are the same as in Fig. S1(a) and the gap is 300 nm long. We see that a transverse displacement away from the axis by up to 100 nm in any direction decreases $\beta_{\rm probe}$ by less than 4%. That is roughly the displacement we deduce for the molecule studied in the main text.

III. MICRO-FLUIDIC CHANNEL FILLING

Fig. S3(a) shows an optical microscope image of unfilled micro-fluidic channels. The channels are 1 µm high and 5 µm wide, tapering to smaller widths in regions where they intersect with the waveguides. Capillary action fills the channels from the edges of the chip with DBT-doped liquid anthracene, which then cools and solidifies. Typically, we find long regions of the solid, separated by shorter voids, as seen in Fig. S3(b). We believe this is due to the formation of separate crystals, which shrink away from each other when the anthracene solidifies, making the transition to higher density.

In Fig. S3(c) we show the image from a scanning confocal fluorescence microscope centred on a segment of a channel that tapers down to a width of 2 µm. Operating at 785 nm wavelength, this image reveals three bright fluorescent centres just outside the taper, which correspond to single DBT molecules inside the channel. On changing the wavelength we find more molecules, some inside the 2 µm-wide section, each of which has a fluorescence spectrum that is typically $100-200$ MHz wide. The blue bars in Fig. S3(d) show a histogram of the resonance linewidths. We repeated these measurements on molecules in a 0.5 µm-wide channel and found the distribution of widths plotted in red in Fig. S3(d). Because the two distributions are essentially identical, we conclude that the width is not due to spectral instability [9] associated with the confining environment, but is simply due to relaxation of the optical dipole through its interaction with thermal phonons. This was to be expected because the temperature of the molecules was 4.7 K - well above the 3 K at which the width normally approaches the limiting value of 40 MHz. In short, the DBT molecules seem well-behaved even inside the small capillaries.

Finally, we varied the angle of the linearly-polarised pump light at low saturation, and recorded the intensity of the fluorescence as a function of the angle. The circles in Fig. S3(e) show data for a typical single molecule, with the solid line showing an excellent fit to the expected $\cos^2(\theta)$ dependence. Here θ is the angle between the laser polarisation and the linear transition dipole-moment of the molecule. In bulk crystals, the orientation of the dipole moment is along the b-axis of the anthracene crystal [10]. We checked to see if the molecules in a 0.5 µm channel are similarly aligned by recording fluorescence spectra of many molecules, each at a range of laser polarisation angles. In Fig. S3(f)

we plot a histogram showing how the orientation of the optical dipoles is distributed around the mean, which we take as 0°. We see that the DBT molecules do indeed have a preferred orientation, which we presume is the b-axis of that particular crystal. This result, together with the good spectral stability, confirms that the melt growth produces crystalline anthracene inside the channels.

IV. IMPERFECT FILTERING OF OFF-RESONANT LIGHT

In Eq. 11 of the main text we give an expression for the power transmitted through the waveguides to the detector, normalised to 1 far from resonance. We reproduce that equation here:

$$T = 1 - \frac{\alpha\beta_{\text{eff}}}{|t_0|}\left\{2\left(\sin(\phi_{\text{T}}) + \frac{\delta\omega}{\Gamma_2}\cos(\phi_{\text{T}})\right) - \frac{\alpha\beta_{\text{eff}}}{|t_0|}\right\}\frac{\Gamma_1/(2\Gamma_2)}{(\delta\omega/\Gamma_2)^2 + 1 + S}\,. \tag{S9}$$

The fourth (i.e. the last) term represents the light at frequency ω that is scattered by the emitter into the probe guide and makes its way through the optics to the detector, which we will call $P(\omega)$. Here we consider the effect of light scattered at other frequencies due to any radiative sidebands that there may be. Let the power reaching the detector in these sidebands be $P(\omega') = \epsilon P(\omega)$. This causes Eq. S9 to become

$$T = 1 - \frac{\alpha\beta_{\text{eff}}}{|t_0|}\left\{2\left(\sin(\phi_{\text{T}}) + \frac{\delta\omega}{\Gamma_2}\cos(\phi_{\text{T}})\right) - \frac{\alpha\beta_{\text{eff}}}{|t_0|}(1+\epsilon)\right\}\frac{\Gamma_1/(2\Gamma_2)}{(\delta\omega/\Gamma_2)^2 + 1 + S}\,, \tag{S10}$$

where the off-resonant light produces no interference term because it is the time-averaged power that we measure. For most applications it will be desirable to have $\epsilon \ll 1$, but even with a good choice of emitter and with filtering of the output, ϵ may well not be zero. In the case of our experiment with the DBT molecule, we estimate that $\epsilon < 5 \times 10^{-2}$. In order to see the effect of this off-resonant light on our determination of β_{eff} and ϕ_{T}, we take a set of values relevant for our experiment: $\alpha = 0.33; \beta_{\text{eff}} = 0.09; |t_0| = 0.7; \phi_{\text{T}} = 61°; \Gamma_1/(2\Gamma_2) = 0.25$. With these values we make a synthetic data set for T using Eq. S10, in which we take $\epsilon = 1$. When we fit Eq. S9 to the synthetic data, taking β_{eff} and ϕ_{T} as the fit parameters, the effect of the greatly exaggerated off-resonant light is to change β from 9% to 8.8% and to change ϕ_{T} from 61° to 60.4°. We conclude that the small amount of off-resonant light that may be reaching the detector in our experiment has a negligible effect on the determination of β_{eff} and ϕ_{T}.

REFERENCES

[1] P. Yao, V. S. Mangarao, and S. Hughes, "On-chip single photon sources using planar photonic crystals and single quantum dots", Laser and Photonics Reviews **4**, 499–516 (2010).

[2] A. Asenjo-Garcia, J. D. Hood, D. E. Chang, and H. J. Kimble, "Atom-light interactions in quasi-one-dimensional nanostructures: A Green's-function perspective", Phys. Rev. A **95**, 033818 (2017).

[3] P. Türschmann, H. Le Jeannic, S. F. Simonsen, H. R. Haakh, S. Götzinger, V. Sandoghdar, P. Lodahl, and N. Rotenberg, "Coherent nonlinear optics of quantum emitters in nanophotonic waveguides", Nanophotonics **8**, 1641–1657 (2019).

[4] D. E. Chang, A. S. Sørensen, E. A. Demler, and M. D. Lukin, "A single-photon transistor using nanoscale surface plasmons", Nature Physics **3**, 807–812 (2007).

[5] D. E. Chang, L. Jiang, A. V. Gorshkov, and H. J. Kimble, "Cavity QED with atomic mirrors", New Journal of Physics **14**, 063003 (2012).

[6] A. Auffeves-Garnier, C. Simon, J.-M. Gerard, and J.-P. Poizat, "Giant Optical Non-linearity induced by a Single Two-Level System interacting with a Cavity in the Purcell Regime", Physical Review A - Atomic, Molecular, and Optical Physics **75**, 053823 (2006).

[7] P. Lodahl, S. Mahmoodian, and S. Stobbe, "Interfacing single photons and single quantum dots with photonic nanostructures", Rev. Mod. Phys. **87**, 347–400 (2015).

[8] A. Javadi, I. Söllner, M. Arcari, S. L. Hansen, L. Midolo, S. Mahmoodian, G. Kiršanskė, T. Pregnolato, E. H. Lee, J. D. Song, S. Stobbe, and P. Lodahl, "Single-photon nonlinear optics with a quantum dot in a waveguide", Nat. Commun. **6**, 8655 (2015).

[9] B. Gmeiner, A. Maser, T. Utikal, S. Götzinger, and V. Sandoghdar, "Spectroscopy and microscopy of single molecules in nanoscopic channels: spectral behavior vs. confinement depth", Phys. Chem. Chem. Phys. **18**, 19588–19594 (2016).

[10] A. A. Nicolet, P. Bordat, C. Hofmann, M. A. Kol'chenko, B. Kozankiewicz, R. Brown, and M. Orrit, "Single dibenzoterrylene molecules in an anthracene crystal: Main insertion sites", ChemPhysChem **8**, 1929–1936 (2007).

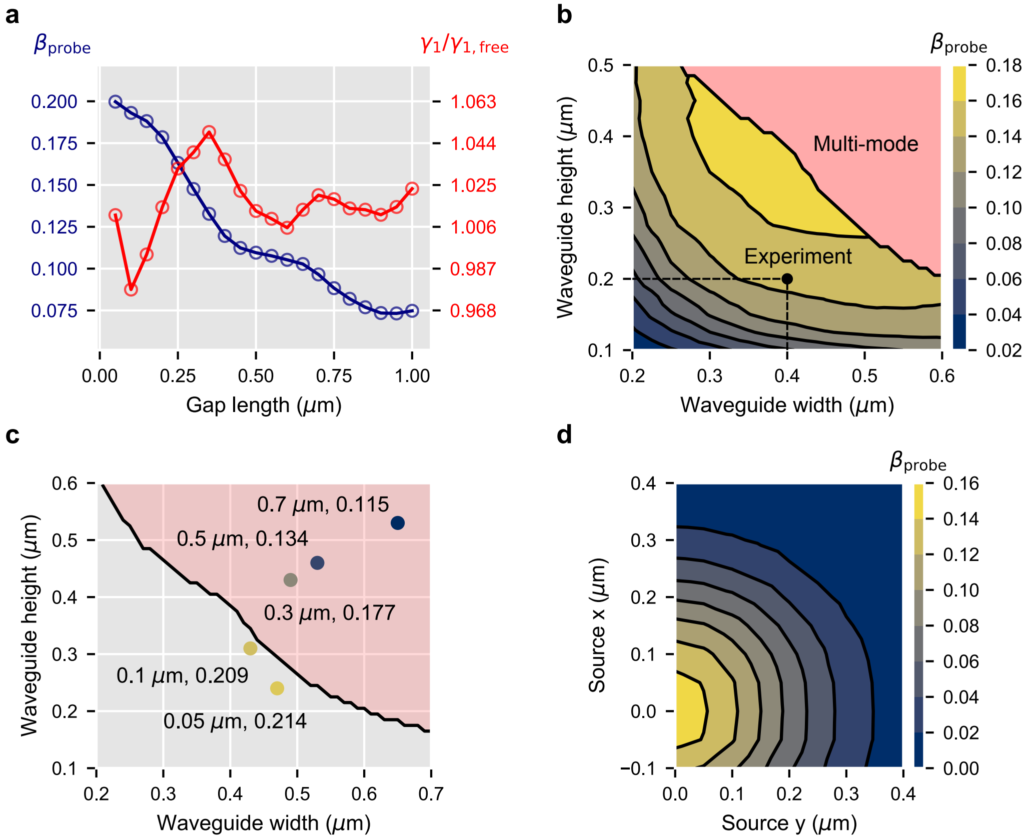

FIG. S1. **FDTD simulations to explore the coupling efficiency in our device.** **a**, One-way efficiency β_{probe} and Purcell factor $\gamma_1/\gamma_{1,\text{free}}$ as a function of gap length. The waveguide is 400 nm wide and 200 nm high. The dipole is polarised along the width of the waveguide and lies in the middle of the gap on the centre line of the waveguide. **b**, With gap length $=$ 300 nm, one-way collection efficiency as a function of waveguide height and width. The waveguide is multi-mode in the pink area. **c**, The position of each point shows the waveguide dimensions that maximise β_{probe} for a given gap length, and the points are labelled by (gap length, β_{probe}). **d**, Efficiency β_{probe} as a function of dipole position in the transverse plane at the centre of the 300 nm gap. Waveguide width $=$ 400 nm, waveguide height $=$ 200 nm.

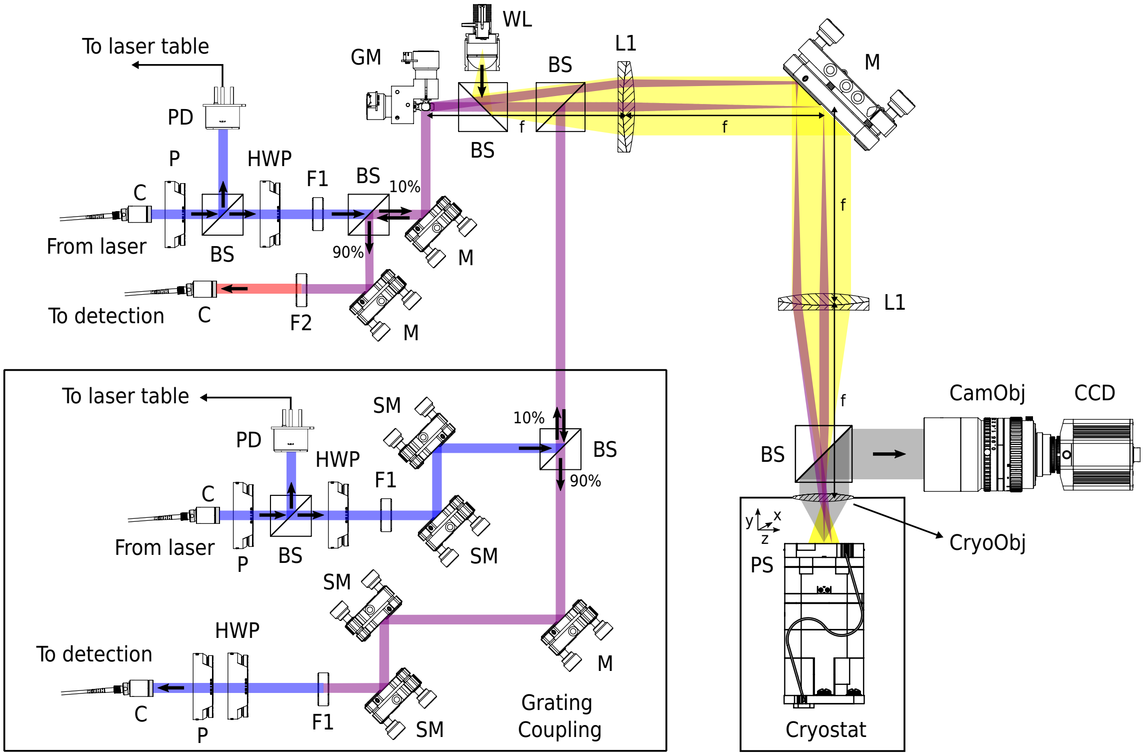

FIG. S2. **Sketch of the fluorescence microscopy set-up.** BS: Beam Splitter, C: Collimator, CamObj: Camera Objective, CryoObj: Cryo objective lens, CCD: Charge-coupled Device, F1: 785 ± 3 nm Band-pass Filter, F2: 800 nm Long-pass Filter, GM: Galvo Mirror, HWP: Half Wave Plate, L1: Achromatic Doublet Lens, M: Mirror, P: Polariser, PD: Photo-Diode, PS: XYZ Position stage, SM: Steering Mirror, WL: White Light.

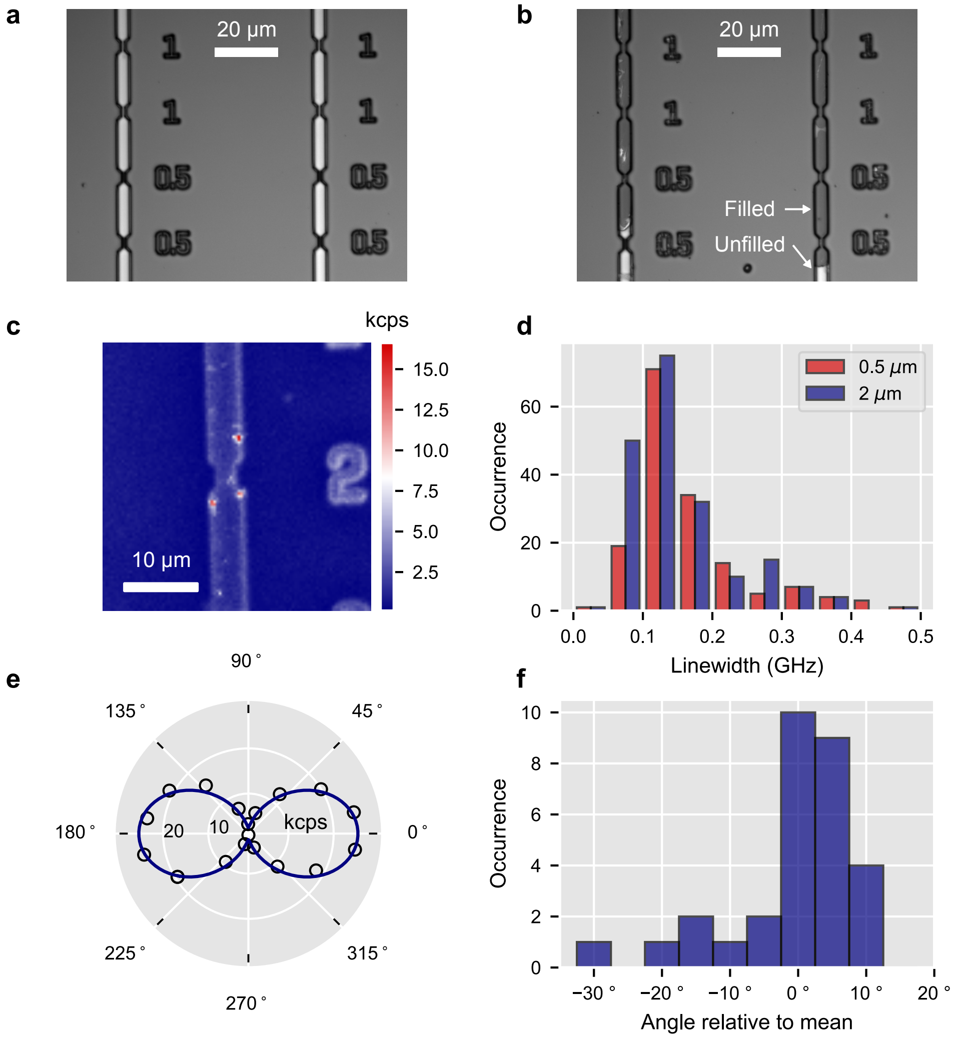

FIG. S3. **Characterisation of filled micro-fluidic channels.** **a**, Optical microscope image of unfilled channels after removal of the sacrificial polymer structures. **b**, Image of the same channels after filling with doped anthracene. **c**, Scanning confocal fluorescence microscopy of a 2 µm channel at 4.7 K with the laser set at 785 nm. **d**, Low-power linewidth distribution of DBT molecules in three 2 µm and three 0.5 µm channels. **e**, Fluorescence intensity at low saturation of a single molecule inside a 0.5 µm channel as a function of excitation laser polarisation. **f**, Histogram of optical dipole moment orientations (projected into the focal plane) in a 0.5 µm channel. The zero is the mean orientation of all the molecule angles.
